# ANGPTL2 Deletion Attenuates Neuroinflammation and Cognitive Dysfunction Induced by Isoflurane in Aged Mice through Modulating MAPK Pathway

**DOI:** 10.1155/2023/2453402

**Published:** 2023-02-21

**Authors:** Xiaoyan Huang, Zegeng Su, Shuncai Zhang, Xiaoling Xu, Bo Yang, Xiang Xu

**Affiliations:** Department of Anesthesiology, Cancer Hospital of Shantou University Medical College, Shantou, Guangdong 515000, China

## Abstract

Postoperative cognitive dysfunction (POCD) is a well-known complication after surgery with cognitive impairments. Angiopoietin-like protein 2 (ANGPTL2) has been found to be associated with inflammation. However, the role of ANGPTL2 in inflammation of POCD is unclear. Here, mice were subjected into isoflurane anesthesia. It was demonstrated that isoflurane increased ANGPTL2 expression and promoted pathological change in brain tissues. However, downregulation of ANGPTL2 alleviated the pathological change and elevated learning and memory abilities, improving isoflurane-induced cognitive dysfunction in mice. In addition, isoflurane-induced cell apoptosis and inflammation were repressed via ANGPTL2 knockdown in mice. Downregulation of ANGPTL2 was also verified to suppress isoflurane-induced microglial activation, evidenced by a decrease of Iba1 and CD86 expressions and an increase of CD206 expression. Further, the isoflurane-induced MAPK signaling pathway was repressed through downregulation of ANGPTL2 in mice. In conclusion, this study proved that downregulation of ANGPTL2 attenuated isoflurane-induced neuroinflammation and cognitive dysfunction in mice via modulating the MAPK pathway, which provided a new therapeutic target for POCD.

## 1. Introduction

Postoperative cognitive dysfunction (POCD) is a commonly seen complication after surgery especially for elderly population [1, 2]. POCD is characterized by cognitive impairments covering short- or long-term memory loss and attention deficit [3]. The morbidity and mortality of POCD are high [4]. POCD was reported to occur in approximately 25.8% shortly postsurgery and in decreasing trend during the following months [5, 6]. The independent risk factors for POCD include increased age, major surgery, history of myocardial infarction, and preexisting cognitive impairment [7, 8]. However, the mechanisms of POCD pathogenesis remain unclear. Thus, it is necessary to investigate its molecular mechanisms for the prevention and treatment of POCD.

Angiopoietin-like protein 2 (ANGPTL2) is one of the eight members of the ANGPTL protein family and is mainly produced by adipose tissues that maintain tissue homeostasis by inducing inflammation and angiogenesis [9, 10]. ANGPTL2 has been reported to regulate chronic inflammation and metabolic abnormalities in obesity, and abnormal expression of ANGPTL2 is associated with multiple tumors [11, 12]. Additionally, ANGPTL2 expression was increased in adipose tissues in high-fat diet-fed mice, and deletion of ANGPTL2 improved adipose tissue inflammation in these mice [9]. ANGPTL2−/− ameliorated acute lung injury progression through downregulation of a number of total cells, total leukocytes in bronchoalveolar lavage fluid, and inflammatory response via inactivating the NF-*κ*B pathway [13]. A previous study has shown that neuroinflammation exerted a key role in the progression of POCD [1]. Moreover, accumulating evidence has indicated that anti-inflammatory therapy could improve POCD [14, 15], suggesting that ANGPTL2 may function in POCD.

In the current study, the aim is to determine how ANGPTL2 could regulate POCD. Mice were subjected into isoflurane anesthesia. The effects of ANGPTL2 on isoflurane-induced cognitive dysfunction and cell inflammation were investigated in mice. This study indicated that ANGPTL2 may provide a new target to improve POCD.

## 2. Materials and Methods

### 2.1. Animals

The ~8-month male C57BL/6 mice were obtained from Experiment Animal Center (Shanghai, China) and assigned into two groups (*n* = 6/group): (A) sham and (B) isoflurane. Animal experiments were carried out in this study in accordance with Guide for the Care and Use of Laboratory Animals and Cancer Hospital of Shantou University Medical College. The mice in the sham group were not exposed to isoflurane. The mice in the isoflurane group were exposed to isoflurane anesthesia in a gas-tight chamber prefilled with 1.5% isoflurane in 100% O_2_ and stayed in the chamber for 2 h. For isoflurane anesthesia, 1.5% isoflurane in 100% O_2_ at 1.5 L/min was continuously gassed to the chamber [16]. Mice were kept to stay in this chamber for 2 h [16]. Finally, the mice were provided with 100% O_2_ for emergency. Additionally, mice were also divided into four groups (*n* = 6/group): (A) sham+Ad-shNC, (B) sham+Ad-shANGPTL2, (C) isoflurane+Ad-shNC, and (D) isoflurane+Ad-shANGPTL2. The mice in the sham+Ad-shANGPTL2 group or isoflurane+Ad-shANGPTL2 were intracerebroventricularly transfected with 15 *μ*L adenovirus-shANGPTL2 (RiboBio, Guangzhou, China) in the left lateral cerebral ventricles. Mice were used for the subsequent experiments after 140 hours.

### 2.2. Hematoxylin and Eosin (HE) Staining

Brain tissues were collected and subjected to fixation. Samples were embedded into wax and sliced into 5 *μ*m. Sections were subjected to deparaffinization and rehydration and stained with hematoxylin and eosin. Finally, the pathological changes were observed through a light microscope.

### 2.3. Western Blot Assay

Proteins were extracted using RIPA lysate (Sangon, Shanghai, China). A bicinchoninic acid (BCA) kit (Boster, Wuhan, China) was used to detect the concentration of protein sample. Samples were added into wells, separated by 10% SDS-PAGE, and transferred onto PVDF membranes (EMD Millipore, Billerica, MA, USA). After blocking in 5% skim milk for 1 h, membranes were incubated with primary antibodies including ANGPTL2 (ab199133, 1 : 1000), cleaved caspase-3 (ab32042, 1 : 800), Bax (ab32503, 1 : 1000), BCL-2 (ab32124, 1 : 1000), p-NF-*κ*B p65 (ab222494, 1 : 1000), NF-*κ*B p65 (ab288751, 1 : 1500), p-ERK1/2 (ab278538, 1 : 1000), ERK1/2 (ab17942, 1 : 1000), p-JNK (ab4821, 1 : 1000), JNK (ab112501, 1 : 1000), p-P38 (ab178867, 1 : 1000), P38 (ab170099, 1 : 1000), and *β*-actin (ab8226, 1 : 3000) (all from Abcam, Cambridge, MA, USA), at 4°C overnight. Secondary antibody (1 : 10000, Cell Signaling, Danvers, MA) was used to incubate the membranes at 37°C for 60 min. Signals were observed using enhanced chemiluminescence (Beyotime, Shanghai, China) with a LAS-3000 imaging system. ImageJ was used for quantitative analysis of the blots.

### 2.4. Quantitative Real-Time PCR (qRT-PCR)

Total RNA from brain tissues were extracted. ReverTra Ace qPCR RT Kit (TIANGEN, Beijing, China) was used to synthesize cDNA. ANGPTL2, TNF-*α*, IL-6, IL-1*β*, CD86, and CD206 mRNA levels were measured using SYBR Premix Ex Taq II (Takara, Japan). *β*-Actin served as an endogenous control. Fold changes were calculated via the 2^−ΔΔCq^ method. The specific primers used for this study are listed in Table 1.

### 2.5. Morris Water Maze Task

The four-quadrant circular pool with a diameter of 160 cm and a height of 50 cm was prepared and filled with regular tap water (22 ± 1°C). In this study, to experience the pool environment, the examined mice were trained with no platform sessions. Subsequently, hidden platform sessions with 1 cm beneath water surface were carried out. Each trial had a limit of 60 s for the escape platform. The trial was stopped when the platform was found. The time to find the platform (escape latency) was recorded. Moreover, the time in target quadrant and the number of platform crossing were also recorded by an automated tracking software. At the start of each trial, mice were placed in the maze from different locations (east, south, west, or north).

### 2.6. Fear Conditioning Test (FCT)

Mice were subjected to fear conditioning test to evaluate learning and memory abilities as previously described [17]. Mice were put in a chamber box with a stainless-steel shock grid floor. Each animal was kept at the chamber for 3 min for adaption and then given two footshocks (0.5 mA; 2 s each time) with an intertribal interval of 1-4 min. After conditioning training, mice were removed from the test chamber for 60 s and subsequently put back to the cage. After 24 h, the contextual fear memory was detected by putting mice in the same chamber for 3 min with no footshock. Total freezing time was recorded and evaluated.

### 2.7. TdT-Mediated dUTP Nick-End Labeling (TUNEL) Staining

The brain tissues were collected and subjected to fixation in formaldehyde. Samples were embedded with paraffin and cut into 5 *μ*m sections. Subsequently, samples were dewaxed and permeabilized with 0.25% Triton-X 100 for 35 min. TUNEL reaction solution (Beyotime, Shanghai, China) was used to cover the sections for 55 min. DAPI was used for nuclear staining. Photographs were captured under a fluorescence microscope.

### 2.8. Enzyme-Linked Immunosorbent Assay (ELISA)

TNF-*α*, IL-6, and IL-1*β* measurement was performed using ELISA. Supernatants from brain tissues were collected. TNF-*α*, IL-6, and IL-1*β* levels were detected, and absorbance value was read at a wavelength of 450 nm.

### 2.9. Immunofluorescence

The brain tissues were collected and fixed in 4% paraformaldehyde. Samples were embedded and sliced into 4 *μ*m thickness. Paraffin-embedded brain tissues were dewaxed and blocked with goat serum. Slides were incubated with Iba1 antibody (Abcam, Cambridge, MA, USA) at 4°C overnight and covered with conjugated secondary antibody for 60 min at 37°C. Nuclei were stained with DAPI for 25 min. A fluorescence microscope was used to observe signals.

### 2.10. Statistical Analyses

Data was expressed as mean ± SD and analyzed using GraphPad Prism 7 software. Differences between two groups were compared with unpaired Student's *t* test. Multiple comparisons were carried out using one-way analysis of variance (ANOVA) by Tukey's. *p* < 0.05 was considered statistically significant.

## 3. Results

### 3.1. Downregulation of ANGPTL2 Improved Isoflurane-Induced Cognitive Dysfunction in Mice

**Figure 1:**
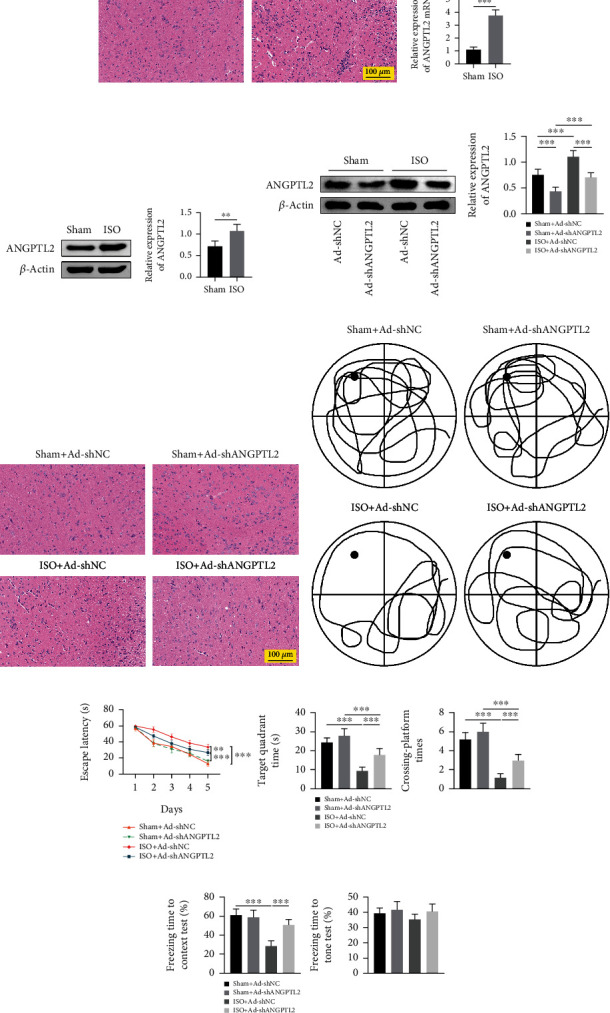
Downregulation of ANGPTL2 improved isoflurane-induced cognitive dysfunction in mice. (a) Pathological change was detected using HE staining. (b) ANGPTL2 mRNA level was measured using qRT-PCR. (c, d) Western blotting was used to examine expression of ANGPTL2 and *β*-actin protein level. (e) HE staining was performed to observe pathological change. (f, g) Morris water maze task was used to detect escape latency time, target quadrant time, and platform crossing time. (h) FCT was carried out to measure freezing time to context test and freezing time to tone test. *n* = 6. ^∗∗^*p* < 0.01; ^∗∗∗^*p* < 0.001.

To investigate the effect of ANGPTL2 on cognitive dysfunction in mice, mice were subjected to isoflurane anesthesia and then brain tissues were collected. HE staining revealed that isoflurane treatment-induced neuronal death increased dead cells with cytoplasmic atrophy and nuclear degeneration (Figure 1(a)). qRT-PCR and western blotting verified that isoflurane elevated ANGPTL2 mRNA level and protein level (Figures 1(b) and 1(c)). Moreover, mice with low ANGPTL2 expression received isoflurane anesthesia and brain tissues were obtained. Western blotting demonstrated that ANGPTL2 was decreased in the sham+Ad-shANGPTL2 group and isoflurane treatment increased ANGPTL2 protein level, whereas ANGPTL2 knockdown inhibited isoflurane-induced ANGPTL2 protein level (Figure 1(d)). HE staining revealed that brain tissues showed no effect on cell damage in the sham+Ad-shANGPTL2 group. Isoflurane treatment enhanced cell damage, whereas ANGPTL2 knockdown alleviated the cell damage (Figure 1(e)). As shown in Figures 1(f) and 1(g), Morris water maze task was conducted and recorded. Results revealed that isoflurane increased escape latency time and decreased target quadrant time and platform crossing time in mice. However, knockdown of ANGPTL2 exerted opposite effects. Additionally, FCT verified that isoflurane decreased percentage of freezing time to context test and had no effect on freezing time to tone test. Conversely, ANGPTL2 knockdown increased the percentage of freezing time in the 24 h context test (Figure 1(h)). The data suggested that downregulation of ANGPTL2 alleviated isoflurane-induced cognitive dysfunction in mice.

### 3.2. Downregulation of ANGPTL2 Inhibited Isoflurane-Induced Cell Apoptosis in Mice

**Figure 2:**
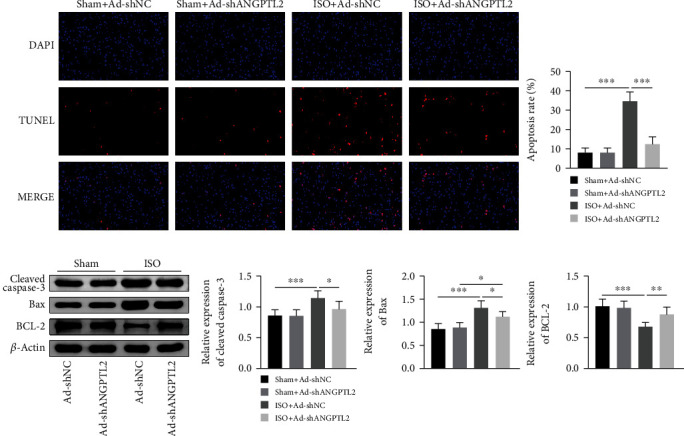
Downregulation of ANGPTL2 inhibited isoflurane-induced cell apoptosis in mice. (a) TUNEL assay was used to detect cell apoptosis of brain tissues. (b) The expression of cleaved caspase-3, Bax, BCL-2, and *β*-actin protein levels was examined via western blotting. *n* = 6. ^∗^*p* < 0.05, ^∗∗^*p* < 0.01, and ^∗∗∗^*p* < 0.001.

We next explored the effect of ANGPTL2 on isoflurane-induced cell apoptosis in mice. TUNEL revealed that isoflurane promoted cell apoptosis in brain tissues, whereas ANGPTL2 knockdown suppressed isoflurane-induced cell apoptosis (Figure 2(a)). Moreover, western blotting demonstrated that isoflurane elevated the expression of cleaved caspase-3 and Bax protein levels and reduced BCL-2 protein level in brain tissues of mice. However, ANGPTL2 knockdown exhibited opposite effects on expression of those proteins (Figure 2(b)). These findings indicated that downregulation of ANGPTL2 inhibited isoflurane-induced cell apoptosis in mice.

### 3.3. Downregulation of ANGPTL2 Suppressed Isoflurane-Induced Inflammation in Mice

**Figure 3:**
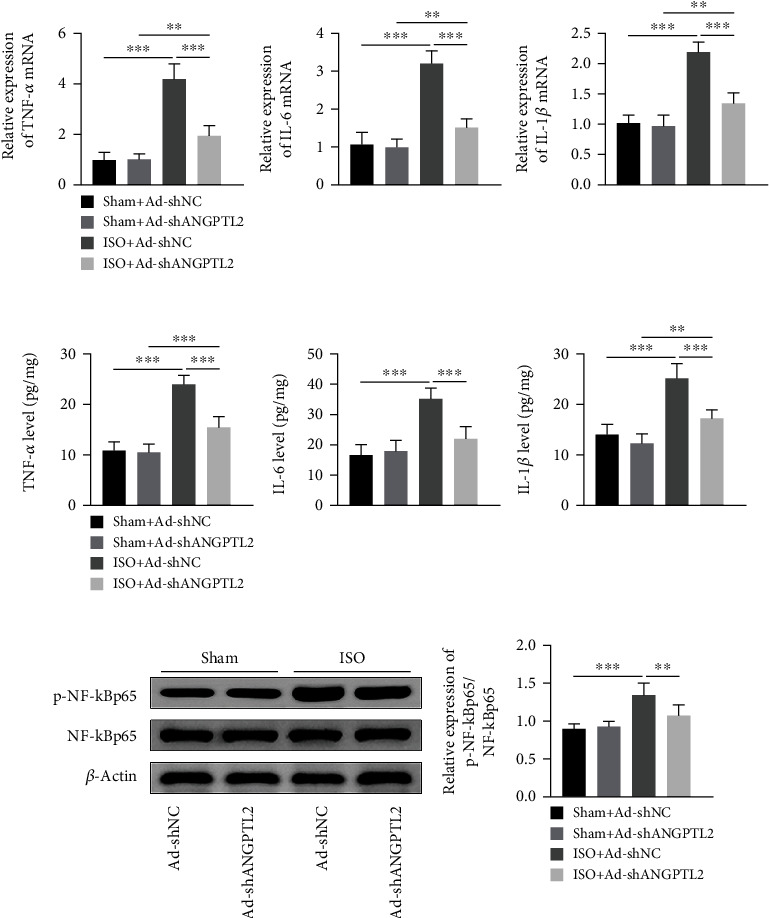
Downregulation of ANGPTL2 suppressed isoflurane-induced inflammation in mice. (a) TNF-*α*, IL-6, and IL-1*β* mRNA levels were detected using qRT-PCR. (b) ELISA was used to examine TNF-*α*, IL-6, and IL-1*β* levels in brain tissues. (c) Western blotting was used to measure p-NF-*κ*B p65, NF-*κ*B p65, and *β*-actin protein levels. *n* = 6. ^∗∗^*p* < 0.01; ^∗∗∗^*p* < 0.001.

To detect the role of ANGPTL2 in isoflurane-induced inflammation in mice, brain tissues were collected. qRT-PCR proved that isoflurane promoted TNF-*α*, IL-6, and IL-1*β* mRNA levels. Conversely, ANGPTL2 knockdown inhibited isoflurane-induced TNF-*α*, IL-6, and IL-1*β* mRNA levels (Figure 3(a)). Consistently, ELISA revealed that isoflurane upregulated TNF-*α*, IL-6, and IL-1*β* levels, whereas ANGPTL2 knockdown exerted opposite effects in the brain tissues of mice (Figure 3(b)). Further, an increase of p-NF-*κ*B p65/NF-*κ*B p65 protein level was induced by isoflurane treatment in the brain tissues of mice. However, ANGPTL2 knockdown reduced isoflurane-induced p-NF-*κ*B p65/NF-*κ*B p65 protein level (Figure 3(c)). These results imply that downregulation of ANGPTL2 suppressed isoflurane-induced inflammation in mice.

### 3.4. Downregulation of ANGPTL2 Decreased Isoflurane-Induced Microglial Activation in Brain Tissues of Mice

**Figure 4:**
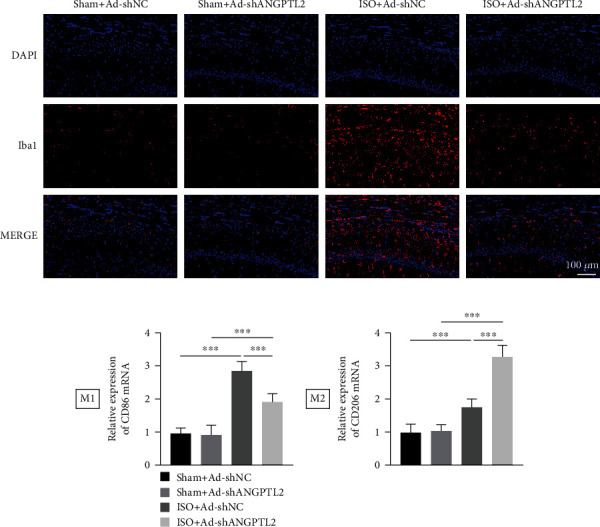
Downregulation of ANGPTL2 decreased isoflurane-induced microglial activation in brain tissues of mice. (a) Immunofluorescence was conducted to examine Iba1 expression. (b) CD86 and CD206 mRNA levels were detected through qRT-PCR. *n* = 6. ^∗∗∗^*p* < 0.001.

We investigated how ANGPTL2 can affect isoflurane-induced microglial activation. Immunofluorescence revealed that isoflurane promoted Iba1 expression, which labelled microglia, whereas ANGPTL2 knockdown inhibited isoflurane-induced Iba1 expression (Figure 4(a)). qRT-PCR demonstrated that isoflurane increased the expression of CD86 and CD206 mRNA levels. However, ANGPTL2 knockdown decreased isoflurane-induced CD86 mRNA level and further promoted isoflurane-induced CD206 mRNA level (Figure 4(b)). These findings suggested that downregulation of ANGPTL2 decreased isoflurane-induced microglial activation in the brain tissues of mice.

### 3.5. Downregulation of ANGPTL2 Inhibited Isoflurane-Induced MAPK Signaling Pathway in Mice

**Figure 5:**
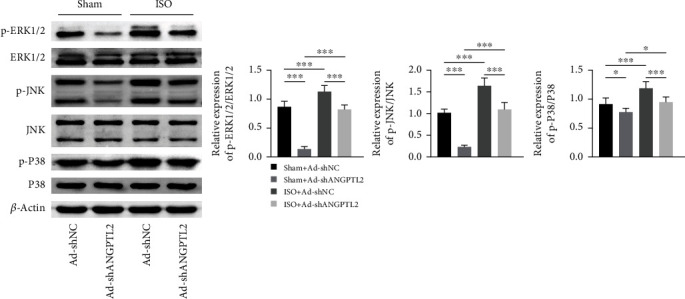
Downregulation of ANGPTL2 inhibited isoflurane-induced MAPK signaling pathway in mice. Western blotting was carried out to measure p-ERK1/2, ERK1/2, p-JNK, JNK, p-P38, P38, and *β*-actin protein levels in brain tissues. *n* = 6. ^∗^*p* < 0.05; ^∗∗∗^*p* < 0.001.

To explore whether ANGPTL2 can inhibit isoflurane-induced MAPK signaling pathway in mice, western blotting was carried out. As shown in Figure 5, results showed that p-ERK1/2/ERK1/2, p-JNK/JNK, and p-P38/P38 protein levels were increased by isoflurane. However, these protein levels were decreased by ANGPTL2 knockdown in mice exposed to isoflurane. The data implied that downregulation of ANGPTL2 inhibited the isoflurane-induced MAPK signaling pathway in mice.

## 4. Discussion

In this study, mice were treated with isoflurane anesthesia. The upregulation of ANGPTL2 was detected in the brain tissues of mice exposed to isoflurane anesthesia. Moreover, downregulation of ANGPTL2 was verified to alleviate the pathological change of brain tissues and improved isoflurane-induced cognitive dysfunction in mice. ANGPTL2 knockdown could also suppress isoflurane-induced cell apoptosis and inflammation and inhibited isoflurane-induced microglial activation. Further, downregulation of ANGPTL2 inhibited isoflurane-induced activation of the MAPK signaling pathway.

Previous studies have proved that isoflurane could contribute to cell apoptosis and was associated with POCD [18, 19]. Anesthesia activated proapoptotic proteins (such as Bax) in the hippocampal region of rats, which may lead to mitochondrial membrane rupture and cystease activation, resulting in apoptosis [20]. Here, this study showed that isoflurane anesthesia induced neuronal death and promoted cognitive dysfunction in mice. Moreover, isoflurane anesthesia increased ANGPTL2 expression, suggesting that ANGPTL2 may be involved in cognitive dysfunction in mice. Thus, we explored the role of ANGPTL2 in mice treated with isoflurane anesthesia. Accumulating evidence has shown that ANGPTL2 was a chronic inflammatory mediator and could be measured in most organs of adult mice [9]. In human macrophage-like cell line, ANGPTL2 overexpression promoted the expression of proinflammatory genes [21]. In ox-LDL-stimulated RAW264.7 cells, upregulation of ANGPTL2 elevated inflammation and cell apoptosis [22]. It is also worth noting that ANGPTL2 knockdown was found to slow atherogenesis in mice via suppressing inflammation and apoptosis in vascular endothelial senescent cells [23]. Interestingly, this research verified that ANGPTL2 knockdown improved isoflurane-induced cognitive dysfunction in mice. Moreover, downregulation of ANGPTL2 repressed isoflurane-induced cell apoptosis in the brain tissues of mice, evidenced by a decreased expression of cleaved caspase-3 and Bax protein levels, as well as an increase of BCL-2 protein level. The decrease of TNF-*α*, IL-6, IL-1*β*, and p-NF-*κ*B p65/NF-*κ*B p65 was caused by ANGPTL2 knockdown, leading to inhibition of cell inflammation in mice. The data indicated that ANGPTL2 exerted an important role in POCD.

Increasing evidence has discovered that strategies based on promoting M1-to-M2 phenotypic conversion of microglial cells could be applied for treating neuroinflammation-induced injury [24]. In this study, ANGPTL2 knockdown suppressed isoflurane-induced Iba1 expression and decreased microglial cells in the brain tissues of mice. Moreover, ANGPTL2 knockdown inhibited isoflurane-induced expression of CD86 (M1 marker) and enhanced isoflurane-induced expression of CD206 (M2 marker). These findings demonstrated that downregulation of ANGPTL2 promoted M1-to-M2 polarization, exerted anti-inflammatory effects, and reduced isoflurane-induced microglial activation in brain tissues of mice. It was also worth noting that ANGPTL2 deletion was found to inhibit osteoclast formation and improve the symptoms of osteoporosis via suppressing the MAPK pathway [25]. In the current study, ANGPTL2 knockdown suppressed isoflurane-induced expression of p-ERK1/2/ERK1/2, p-JNK/JNK, and p-P38/P38 protein levels, leading to inhibition of the MAPK signaling pathway in the brain tissues of mice. Notably, cognitive dysfunction and brain tissue damage were affected in the sham+Ad-shANGPTL2 group, whereas the MAPK signaling pathway was suppressed, suggesting that ANGPTL2 may function in the MAPK signaling pathway easily and rapidly when mice were not exposed to isoflurane. These results implied that ANGPTL2 functioned in neuroinflammation and cognitive dysfunction through the MAPK signaling pathway.

However, previous studies have shown that the PI3K signaling pathway has been found to be involved in regulating cognitive dysfunction [26–28], suggesting that we need to further explore more underlying mechanism of the role of ANGPTL2 in isoflurane-induced neuroinflammation and cognitive dysfunction in the future. Moreover, how ANGPTL2 function in clinical samples is still unclear. Therefore, more research is needed to be performed in the rear future.

In summary, our findings above demonstrated that ANGPTL2 knockdown ameliorated isoflurane-induced cognitive dysfunction and inhibited isoflurane-induced cell apoptosis, inflammation, and microglial activation in the brain tissues of mice through inactivating the MAPK signaling pathway, which may provide a novel potential target for POCD treatment.

## Figures and Tables

**Table 1:** The primer sequences in this study.

Name	Primer sequences (5′-3′)
ANGPTL2	F: CGCCTGGATGGCTCTGTC
	R: GTTTGTAGTTGCCTTGGTTCGTG
TNF-*α*	F: GCATGATCCGAGATGTGGAACTGG
	R: CGCCACGAGCAGGAATGAGAAG
IL-6	F: AGGAGTGGCTAAGGACCAAGACC
	R: TGCCGAGTAGACCTCATAGTGACC
IL-1*β*	F: ATCTCACAGCAGCATCTCGACAAG
	R: CACACTAGCAGGTCGTCATCATCC
CD86	F: TAGGGATAACCAGGCTCTAC
	R: CGTGGGTGTCTTTTGCTGTA
CD206	F: AGTTGGGTTCTCCTGTAGCCCAA
	R: ACTACTACCTGAGCCCACACCTGCT
*β*-Actin	F: CCCATCTATGAGGGTTACGC
	R: TTTAATGTCACGCACGATTTC

## Data Availability

All data generated or analyzed during this study are included in this published article.
